# Efficient and Accurate Optimal Linear Phase FIR Filter Design Using Opposition-Based Harmony Search Algorithm

**DOI:** 10.1155/2013/320489

**Published:** 2013-06-10

**Authors:** S. K. Saha, R. Dutta, R. Choudhury, R. Kar, D. Mandal, S. P. Ghoshal

**Affiliations:** ^1^Department of ECE, NIT Durgapur, Durgapur 713209, India; ^2^Department of ECE, BCET, Durgapur, India; ^3^Department of EE, NIT Durgapur, Durgapur 713209, India

## Abstract

In this paper, opposition-based harmony search has been applied for the optimal design of linear phase FIR filters. RGA, PSO, and DE have also been adopted for the sake of comparison. The original harmony search algorithm is chosen as the parent one, and opposition-based approach is applied. During the initialization, randomly generated population of solutions is chosen, opposite solutions are also considered, and the fitter one is selected as *a priori* guess. In harmony memory, each such solution passes through memory consideration rule, pitch adjustment rule, and then opposition-based reinitialization generation jumping, which gives the optimum result corresponding to the least error fitness in multidimensional search space of FIR filter design. Incorporation of different control parameters in the basic HS algorithm results in the balancing of exploration and exploitation of search space. Low pass, high pass, band pass, and band stop FIR filters are designed with the proposed OHS and other aforementioned algorithms individually for comparative optimization performance. A comparison of simulation results reveals the optimization efficacy of the OHS over the other optimization techniques for the solution of the multimodal, nondifferentiable, nonlinear, and constrained FIR filter design problems.

## 1. Introduction

Digital filter is essentially a system or network that improves the quality of a signal and/or extracts information from signals or separates two or more signals which are previously combined. The linear time invariant (LTI) system and the filter are synonymous and are often used to perform spectral shaping or frequency selective filtering. The nature of this filtering action is determined by the frequency response characteristics, which depend on the choice of system parameters, that is, the coefficients of the difference equations. Thus, by proper selection of the coefficients, one can design frequency selective filters that pass signals with frequency components in some bands while attenuate signals containing frequency components in other frequency bands [1, 2]. There are different techniques for the design of FIR filters, such as window method and frequency sampling method. All these methods are based on approximation to the frequency characteristics of ideal filters. The design method is based on the requirements of ripples in the passband and the stopband, the stop band attenuation and the transition width. In the window method, ideal impulse response is multiplied with a window function. There are various kinds of window functions (Butterworth, Chebshev, Kaiser, etc.). These windows limit the infinite length impulse response of ideal filter into a finite window to design an actual response [3–5]. But the major drawback of windowing methods is that it does not allow sufficient control of the frequency response in the various frequency bands and other filter parameters such as transition width, and it tends to process relatively long filter lengths. The designer always has to compromise on one or other design specifications [6]. The conventional gradient-based optimization method [7] and other classical optimization algorithms [3, 4] are not sufficient to optimize multimodal and nonuniform objective functions of FIR filters, and the objective function cannot converge to the global minimum solution. So, evolutionary methods have been implemented in the design of optimal digital filters with better control of filter parameters and achievement of the highest stop band attenuation and the lowest stop band ripples.

Different evolutionary optimization techniques are reported in the literatures. When considering global optimization methods for digital filter design, GA [8–11] seems to have attracted a considerable attention. Although standard GA (also known as real-coded GA (RGA)) shows a good performance for finding the promising regions of the search space, they are inefficient in determining the global optimum and prone to revisiting the same suboptimal solution. In order to overcome the problem associated with RGA, orthogonal genetic algorithm (OGA) [12], hybrid Taguchi GA (TGA) [13] have been proposed. Tabu search [14], Simulated Annealing (SA) [15, 16], Bee Colony algorithm (BCA) [17], differential evolution (DE) [18, 19], seeker optimization algorithm [20], particle swarm optimization (PSO) [21–23], opposition-based BAT (OBAT) algorithm [24], some variants of PSO like PSO with Quantum Infusion (PSO-QI) [25, 26], adaptive inertia weight PSO [27], chaotic mutation PSO (CMPSO) [28, 29], Novel PSO (NPSO) [30], Gravitational search algorithm (GSA) [31], seeker optimization algorithm (SOA) [32], some hybrid algorithms like DE-PSO [33] have also been used for the filter design problems with varying degree of comparative optimization effectiveness. 

Most of the above algorithms show the problems of fixing algorithm's control parameters, premature convergence, stagnation, and revisiting of the same solution over and again. In order to overcome these problems, in this paper, a novel optimization algorithm called opposition-based harmony search (OHS) is employed for the FIR filter design. 

Tizhoosh introduced the concept of *opposition-based learning *(OBL) in [34]. This notion has been applied to accelerate the reinforcement learning [35–37] and the back propagation learning [38] in neural networks. The main idea behind OBL is the simultaneous consideration of an estimate and its corresponding opposite estimate (i.e., guess and opposite guess) in order to achieve a better approximation for the current candidate solution. In the recent literature, the concept of opposite numbers has been utilized to speed up the convergence rate of an optimization algorithm, for example, opposition-based differential evolution (ODE) [39]. Can this idea of opposite number OBL be incorporated during the harmony memory (HM) initialization and also for generating the new harmony vectors during the process of HS? In this paper, OBL has been utilized to accelerate the convergence rate of the HS. Hence, our proposed approach has been called as opposition-based HS (OHS). OHS uses opposite numbers during HM initialization and also for generating the new HM during the evolutionary process of HS.

This paper describes the comparative optimal designs of linear phase low pass (LP), high pass (HP), band pass (BP), and band stop (BS) FIR digital filters using other aforementioned algorithms and the proposed OHS approach individually. The OHS does not prematurely restrict the searching space. A comparison of optimal designs reveals better optimization efficacy of the proposed algorithm over the other optimization techniques for the solution of the multimodal, nondifferentiable, highly nonlinear, and constrained FIR filter design problems. 

The rest of the paper is arranged as follows. In Section 2, the FIR filter design problem is formulated.  Section 3 briefly discusses the evolutionary approaches employed for the FIR filter designs.  Section 4 describes the simulation results obtained by employing PM, RGA, PSO, DE, and OHS. Finally, Section 5 concludes the paper.

## 2. FIR Filter Design

Digital filters are classified as finite impulse response (FIR) or infinite impulse response (IIR) filter depending upon whether the response of the filter is dependent on only the present and past inputs or on the present and past inputs as well as previous outputs, respectively.

An FIR filter has a system function of the form given in the following:

(1) $$\begin{array}{c} H\left(z\right)=h\left(0\right)+h\left(1\right)z^{-1}+\cdots +h\left(N\right)z^{-N} \end{array}$$

or

(2) $$\begin{array}{c} H\left(z\right)=\sum_{n=0}^{N}h\left(n\right)z^{-n}, \end{array}$$

where *h*(*n*) is called impulse response.

The difference equation representation is

(3) $$\begin{array}{c} y\left(n\right)=h\left(0\right)x\left(n\right)+h\left(1\right)x\left(n-1\right)+\cdots +h\left(N\right)x\left(x-N\right). \end{array}$$

The order of the filter is *N*, while the length of the filter (which is equal to the number of coefficients) is *N* + 1. The FIR filter structures are always stable and can be designed to have linear phase response. The impulse responses *h*(*n*) are to be determined in the design process and the values of *h*(*n*) will determine the type of the filter, for example, LP, HP, BP, BS, and so forth. The choice of filters is based on four broad criteria. The filters should provide the following: as little distortion as possible to the signal; flat pass band and exhibit high attenuation characteristics with as low as possible stop band ripples. 

Other desirable characteristics include short filter length, short frequency transition beyond the cut-off point, and the ability to manipulate the attenuation in the stop band. In many filtering applications −3 dB frequency *f*_−3dB_ has become a recognizable parameter for defining the cut-off frequency *f*_*c*_ (the frequency at which the magnitude attains an absolute value of 0.5). The consequence of using the 3 dB measure is that it varies with filter length since the sharpness of the transition width is a function of the filter order. Additionally, as the filter order increases, the transition width decreases, and *f*_−3 dB_ approaches *f*_*c*_ asymptotically [40, 41]. In any filter design problem, some of these parameters are fixed while others are optimized.

In this paper, the OHS is applied in order to obtain the actual filter response as close as possible to the ideal filter response. The designed FIR filter with *h*(*n*) individuals or particles/solutions is positive even symmetric and of even order. The length of *h*(*n*) is *N* + 1; that is, the number of coefficients is also *N* + 1. In each iteration, these solutions are updated. Fitness values of updated solutions are calculated using the new coefficients and the new error fitness function. The solution obtained after a certain number of iterations or after the error fitness below a certain limit is considered to be the optimal result. The error is used to evaluate the error fitness function of the solution. It takes the error between the magnitudes of frequency responses of the ideal and the actual filters. An ideal filter has a magnitude of one on the pass band and a magnitude of zero on the stop band. The error fitness function is minimized using the evolutionary algorithms RGA, PSO, DE, and OHS, individually. The individuals that have lower error fitness values represent the better filter, that is, the filter with better frequency response.

The frequency response of the FIR digital filter can be calculated as

(4) $$\begin{array}{c} H\left(e^{jw_{k}}\right)=\sum_{n=0}^{N}h\left(n\right)e^{-jw_{k}n}, \end{array}$$

where *ω*_*k*_ = 2*πk*/*N* and *H*(*e* ^*jw*_*k*_^) or *H*(*w*_*k*_) is the Fourier transform complex vector. The frequency is sampled with *N* points in [0, *π*]. One has the following:

(5) Hd(ω)=[Hd(ω1),Hd(ω2),Hd(ω3),…,Hd(ωN)]T, Hi(ω)=[Hi(ω1),Hi(ω2),Hi(ω3),…,Hi(ωN)]T,

where *H*_*i*_ represents the magnitude response of the ideal filter and for LP, HP, BP, and BS, it is given, respectively, as

(6) Hi(ωk)={1for  0≤ω≤ωc;0otherwiseHi(ωk)={0for  0≤ω≤ωc;1otherwiseHi(ωk)={1for  ωpl≤ω≤ωph;0otherwiseHi(ωk)={0for  ωpl≤ω≤ωph;1otherwise

*H*_*d*_(*ω*_*k*_) represents the approximate actual filter to be designed, and the *N* is the number of samples.

 Different kinds of fitness functions have been used in different literatures as given in the following [18, 19, 21]

(7) $$\begin{array}{c} \text{Error}=\max\left\{\sum_{i=1}^{N}\left[\left|\left|H_{d}\left(\omega_{k}\right)\right|-\left|H_{i}\left(\omega_{k}\right)\right|\right|\right]\right\}, \end{array}$$

(8) $$\begin{array}{c} \text{Error}={\left\{\sum_{i=1}^{N}{\left[\left|H_{d}\left(\omega_{k}\right)\right|-\left|H_{i}\left(\omega_{k}\right)\right|\right]}^{2}\right\}}^{1/2}. \end{array}$$

An error function given by the following equation is the approximate error function used in popular Parks-McClellan (PM) algorithm for digital filter design [3]:

(9) $$\begin{array}{c} E\left(\omega \right)=G\left(\omega \right)\left[H_{d}\left(\omega_{k}\right)-H_{i}\left(\omega_{k}\right)\right], \end{array}$$

where *G*(*ω*) is the weighting function used to provide different weights for the approximate errors in different frequency bands.

The major drawback of the PM algorithm is that the ratio of *δ*_*p*_/*δ*_*s*_ is fixed. In order to improve the flexibility in the error function to be minimized so that the desired levels of *δ*_*p*_ and *δ*_*s*_ may be individually specified, the error function given in the following equation has been considered as fitness function in [23, 26]:

(10) $$\begin{array}{c} J_{1}=\underset{\omega \leq \omega_{p}}{\max}\left(\left|E\left(\omega \right)\right|-\delta_{p}\right)+\underset{\omega \geq \omega_{s}}{\max}\left(\left|E\left(\omega \right)\right|-\delta_{s}\right), \end{array}$$

where *δ*_*p*_ and *δ*_*s*_ are the ripples in the pass band and the stop band, respectively and *ω*_*p*_ and *ω*_*s*_ are the pass band and stop band normalized edge frequencies, respectively.

In this paper, a novel error fitness function has been adopted in order to achieve higher stop band attenuation and to have moderate control on the transition width. The error fitness function used in this paper is given in (11). Using the following equation, it is found that the proposed filter design approach results in considerable improvement over the PM and other optimization techniques:

(11) $$\begin{array}{c} J_{2}=\sum_{}\text{abs}\left[\text{abs}\left(\left|H\left(\omega \right)\right|-1\right)-\delta_{p}\right]+\sum \left[\text{abs}\left(\left|H\left(\omega \right)\right|-\delta_{s}\right)\right]. \end{array}$$

For the first term of (11), *ω*∈ pass band including a portion of the transition band, and for the second term of (11), *ω*∈ stop band including the rest portion of the transition band. The portions of the transition band chosen depend on pass band edge and stop band edge frequencies.

The error fitness function given in (11) represents the generalized fitness function to be minimized using the evolutionary algorithms, individually. Each algorithm individually tries to minimize this error and thus improves the filter performance. Since the coefficients of the linear phase FIR filter are matched, the dimension of the problem is thus halved. By only determining half of the coefficients, the FIR filter can be designed. This greatly reduces the computational burdens of the algorithms applied to the design of linear phase FIR filters.

## 3. Optimization Techniques Employed

Evolutionary algorithms stand upon some common characteristics like stochastic, adaptive, and learning in order to produce intelligent optimization schemes. Such schemes have the potential to adapt to their ever-changing dynamic environment through the previously acquired knowledge. Tizhoosh introduced the concept of opposition-based learning (OBL) in [34]. In this paper, OBL has been utilized to accelerate the convergence rate of the HS. Hence, our proposed approach has been called as opposition-based harmony search (OHS). OHS uses opposite numbers during HM initialization and also for generating the new harmony memory (HM) during the evolutionary process of HS. The other algorithms RGA, PSO, and DE considered in this paper are well known and not discussed here.

### 3.1. A Brief Description of HS Algorithm

In the basic HS algorithm, each solution is called a *harmony*. It is represented by an *n*-dimension real vector. An initial randomly generated population of harmony vectors is stored in an HM. Then, a new candidate harmony is generated from all the solutions in the HM by adopting a memory consideration rule, a pitch adjustment rule, and a random reinitialization. Finally, the HM is updated by comparing the new candidate harmony vector and the worst harmony vector in the HM. The worst harmony vector is replaced by the new candidate vector if it is better than the worst harmony vector in the HM. The above process is repeated until a certain termination criterion is met. Thus, the basic HS algorithm consists of three basic phases. These are initialization, improvisation of a harmony vector, and updating the HM. Sequentially, these phases are described below.

#### 3.1.1. Initialization of the Problem and the Parameters of the HS Algorithm

In general, a global optimization problem can be enumerated as follows: min⁡ *f*(*x*) s.t. *x*_*j*_ ∈ [*para*_*j*_^min⁡^, *para*_*j*_^max⁡^], *j* = 1, 2,…, *n* where *f*(*x*) is the objective function, *X* = [*x*_1_, *x*_2_,…, *x*_*n*_] is the set of design variables, and *n* is the number of design variables. Here, *para*_*j*_^min⁡^, *para*_*j*_^max⁡^are the lower and upper bounds for the design variable *x*_*j*_, respectively. The parameters of the HS algorithm are the harmony memory size (*HMS*) (the number of solution vectors in HM), the harmony memory consideration rate (*HMCR*), the pitch adjusting rate (*PAR*), the distance bandwidth (*BW*), and the number of improvisations (*NI*); *NI* is the same as the total number of fitness function calls (NFFCs). It may be set as a stopping criterion.

#### 3.1.2. Initialization of the HM

The HM consists of *HMS* harmony vectors. Let *X*_*j*_ = [*x*_1_^*j*^, *x*_2_^*j*^,…, *x*_*n*_^*j*^] represent the *j*th harmony vector, which is randomly generated within the parameter limits [*para*_*j*_^min⁡^, *para*_*j*_^max⁡^]. Then, the HM matrix is filled with the *HMS* harmony vectors as in the following:

(12) $$\begin{array}{c} \text{HM}=\left[\begin{array}{cccc} x_{1}^{1} & x_{2}^{1} & \cdots & x_{n}^{1}\\ x_{1}^{2} & x_{2}^{2} & \cdots & x_{n}^{2}\\ \cdots & & & \\ x_{1}^{HMS} & x_{2}^{HMS} & \cdots & x_{n}^{HMS} \end{array}\right]. \end{array}$$

#### 3.1.3. Improvisation of a New Harmony

A new harmony vector *X* ^new^ = (*x*_1_^new^, *x*_2_^new^ ⋯ *x*_*n*_^new^) is generated (called improvisation) by applying three rules, namely, (i) a memory consideration, (ii) a pitch adjustment, and (iii) a random selection. First of all, a uniform random number *r*_1_ is generated in the range [0, 1]. If *r*_1_ is less than *HMCR*, the decision variable *x*_*j*_^new^ is generated by the memory consideration; otherwise, *x*_*j*_^new^ is obtained by a random selection (i.e., random reinitialization between the search bounds). In the memory consideration, *x*_*j*_^new^ is selected from any harmony vector *i* in [1, 2,…*HMS*]. Secondly, each decision variable *x*_*j*_^new^ will undergo a pitch adjustment with a probability of *PAR* if it is updated by the memory consideration. The pitch adjustment rule is given as follows

(13) $$\begin{array}{c} x_{j}^{\text{new}}=x_{j}^{\text{new}}\pm r_{3}\times BW, \end{array}$$

where *r*_3_ is a uniform random number between 0 and 1.

#### 3.1.4. Updating of HM

After a new harmony vector *X*_*j*_^new^ is generated, the HM will be updated by the survival of the fitter vector between *X* ^new^ and the worst harmony vector *X* ^worst^ in the HM. That is, *X* ^new^ will replace *X* ^worst^ and become a new member of the HM if the fitness value of *X* ^new^ is better than the fitness value of *X* ^worst^.

The computational procedure of the basic HS algorithm can be summarized as shown in Algorithm 1.

### 3.2. The Improved Harmony Search (IHS) Algorithm

The basic HS algorithm uses fixed values for *PAR* and *BW* parameters. The IHS algorithm, proposed by Mahdavi et al. [42], applies the same memory consideration, pitch adjustment, and random selection as the basic HS algorithm, but dynamically updates the values of *PAR* and *BW* as in (14) and (15), respectively:

(14) $$\begin{array}{c} PAR\left(gn\right)=PAR^{\min}+\frac{PAR^{\max}-PAR^{\min}}{NI}\times gn, \end{array}$$

(15) $$\begin{array}{c} BW\left(gn\right)=BW^{\max}\times e^{\left((\ln(\left({BW}^{\min})/({BW}^{\max}\right))/NI)\times gn\right)}. \end{array}$$

In (14), *PAR*(*gn*) is the pitch adjustment rate in the current generation (*gn*); *PAR* ^min⁡^ and *PAR* ^max⁡^ are the minimum and the maximum adjustment rates, respectively. In (15), *BW*(*gn*) is the distance bandwidth at generation (*gn*); *BW* ^min⁡^ and *BW* ^max⁡^ are the minimum and the maximum bandwidths, respectively.

### 3.3. Opposition-Based Learning: A Concept

Evolutionary optimization methods start with some initial solutions (initial population) and try to improve them toward some optimal solution(s). The process of searching terminates when some predefined criteria are satisfied. In the absence of *a priori *information about the solution, we usually start with random guesses. The computation time, among others, is related to the distance of these initial guesses from the optimal solution. We can improve our chance of starting with a closer (fitter) solution by simultaneously checking the opposite solution [34]. By doing this, the fitter one (guess or opposite guess) can be chosen as an initial solution. In fact, according to the theory of probability, 50% of the time a guess is further from the solution than its opposite guess [36]. Therefore, starting with the closer of the two guesses (as judged by its fitness) has the potential to accelerate convergence. The same approach can be applied not only to initial solutions but also continuously to each solution in the current population [36].

#### 3.3.1. Definition of Opposite Number

 Let *x* ∈ [*ub*, *lb*] be a real number. The opposite number is defined as

(16) $$\begin{array}{c} \overset{˘}{x}=ub+lb-x. \end{array}$$

Similarly, this definition can be extended to higher dimensions [34] as stated in the next subsection.

#### 3.3.2. Definition of Opposite Point 

 Let *X* = (*x*_1_, *x*_2_,…, *x*_*n*_) be a point in *n*-dimensional space, where (*x*_1_, *x*_2_,…, *x*_*n*_) ∈ *R* and *x*_*i*_ ∈ [*ub*_*i*_, *lb*_*i*_] for  all *i*∈{1, 2,…, *n*}. The opposite point $\overset{˘}{X}=\left({\overset{˘}{x}}_{1},{\overset{˘}{x}}_{2}\ldots {,\overset{˘}{x}}_{n}\right)$ is completely defined by its components as

(17) $$\begin{array}{c} {\overset{˘}{x}}_{i}=ub_{i}+lb_{i}-x_{i}. \end{array}$$

Now, by employing the opposite point definition, the opposition-based optimization is defined in the following subsection.

#### 3.3.3. Opposition-Based Optimization

Let *X* = (*x*_1_, *x*_2_,…, *x*_*n*_) be a point in *n*-dimensional space (i.e., a candidate solution). Assume *f* = (·) is a fitness function which is used to measure the candidate's fitness. According to the definition of the opposite point, $\overset{˘}{X}=({\overset{˘}{x}}_{1},{\overset{˘}{x}}_{2},\ldots ,{\overset{˘}{x}}_{n})$ is the opposite of *X* = (*x*_1_, *x*_2_,…, *x*_*n*_). Now, if $f(\overset{˘}{X})\geq f(X)$, then point *X* can be replaced with $\overset{˘}{X}$; otherwise, we continue with *X*. Hence, the point and its opposite point are evaluated simultaneously in order to continue with the fitter one.

### 3.4. Opposition-Based Harmony Search (OHS) Algorithm

Similar to all population-based optimization algorithms, two main steps are distinguishable for HS, namely, HM initialization and producing new HM by adopting the principle of HS. In the present work, the strategy of the OBL [34] is incorporated in those two steps. The original HS is chosen as a parent algorithm, and opposition-based ideas are embedded in it with an intention to exhibit accelerated convergence profile. Corresponding pseudo code for the proposed OHS approach can be summarized as shown in Algorithm 2.

## 4. Results and Discussions

This section presents the simulations performed in MATLAB 7.5 for the design of LP, HP, BP, and BS FIR filters. Each filter order (*N*) is taken as 20, which results in the number of coefficients as 21. The sampling frequency is taken to be *f*_*s*_ = 1 Hz. The number of frequency samples is 128. Each algorithm is run for 50 times to obtain its best results.  Table 1 shows the best chosen control parameters for RGA, PSO, DE and OHS, respectively. 

The parameters of the filters to be designed using any algorithm are as follows: pass band ripple (*δ*_*p*_) = 0.1, stop band ripple (*δ*_*s*_) = 0.01. For the LP filter, pass band (normalized) edge frequency (*ω*_*p*_) = 0.45; stop band (normalized) edge frequency (*ω*_*s*_) = 0.55; transition width = 0.1. For the HP filter, stop band (normalized) edge frequency (*ω*_*s*_) = 0.45; pass band (normalized) edge frequency (*ω*_*p*_) = 0.55; transition width = 0.1. For the BP filter, lower stop band (normalized) edge frequency (*ω*_*sl*_) = 0.25; lower pass band (normalized) edge frequency (*ω*_*pl*_) = 0.35; upper pass band (normalized) edge frequency (*ω*_*ph*_) = 0.65; upper stop band (normalized) edge frequency (*ω*_*sh*_) = 0.75; transition width = 0.1. For the BS filter, lower pass band (normalized) edge frequency (*ω*_*pl*_) = 0.25; lower stop band (normalized) edge frequency (*ω*_*sl*_) = 0.35; upper stop band (normalized) edge frequency (*ω*_*sh*_) = 0.75; upper pass band (normalized) edge frequency (*ω*_*ph*_) = 0.85; transition width = 0.1. Tables 2, 3, 4, and 5 show the optimized filter coefficients obtained for LP, HP, BP, and BS FIR filters, respectively, using RGA, PSO, DE, and OHS, individually. 

Table 6 shows the highest stop band attenuations for all four types of filters using OHS as 35.16 dB (for LP filter), 33.86 dB (for HP filter), 34.76 dB (for BP filter), and 32.45 dB (for BS filter) as compared to those of PM, RGA, PSO and DE. Tables 7, 8, 9 and 10 show the comparative results of performance parameters in terms of maximum and average stop band ripple (normalized), transition width (normalized) for LP, HP, BP and BS filters using PM, RGA, PSO, DE and OHS, respectively. It is also noticed that for almost same level of transition width and stop band ripple, OHS results in the best stop band attenuation among all algorithms for all types of filters. Tables 11, 12, 13, and 14 summarize maximum, mean, variance, and standard deviation for pass band ripple (normalized) and stop band attenuations in dB for LP, HP, BP and BS filters using all concerned algorithms.

In Table 15, OHS-based results are compared with other reported results. Oliveira et al. [15] have designed 30th-order BP filter with stop band attenuation and transition width of 33 dB and 0.1, respectively. A 20th-order LP filter has been designed by Karaboga and Cetinkaya [18] with transition width, pass band, and stop band ripples of 0.16, 0.08, and 0.09, respectively. Liu et al. [19] also reported for 20th-order FIR filter with transition width, pass band and stop band ripples of 0.06, 0.04, and 0.07, respectively. Najjarzadeh and Ayatollahi [21] have designed LP and BP filters of order 33 with approximate values of stop band attenuation 29 dB and 25 dB, respectively. A 30th-order FIR filter has been designed by Ababneh and Bataineh [23] with stop band attenuation, transition width, pass band, and stop band ripples of 30 dB, 0.05, 0.15, and 0.031, respectively. Sarangi et al. [26] have designed 20th-order FIR filters with stop band attenuation, transition width, pass band, and stop band ripples for LP filter of values 27 dB, 0.15, 0.1, and 0.06, respectively. For the same order BP these values are, respectively, as follows: 8 dB, 0.07, 0.2, and 0.05. Mondal et al. have reported the 20th-order HP filter [30] with stop band attenuation, transition width, pass band and stop band ripples of 34.03 dB, 0.0825, 0.129, and 0.02392, respectively. Luitel and Venayagamoorthy also have designed 20th-order LP filter with stop band attenuation, transition width, pass band and stop band ripples of 27 dB, 0.13, 0.291, and 0.270, respectively, as reported in [33].

In this paper OHS-based design is applied to 20th-order LP, HP, BP, and BS filters. maximum stop band attenuations of 35.16 dB, 33.86 dB, 34.76 dB, and 32.45 dB; maximum pass band ripples of 0.140, 0.140, 0.153, and 0.140; Maximum stop band ripples of 0.01746, 0.02027, 0.01828, and 0.02385; Transition width values of 0.0994, 0.1004, 0.0988, and 0.1069 are achieved, respectively, with LP, HP, BP, and BS filters. The above-mentioned values can be verified from the results presented in Table 15. Thus, it is observed from Table 15 that the stop band attenuations in all cases for the 20th-order filters using OHS are much better than the other reported results. 

**Figure 1:**
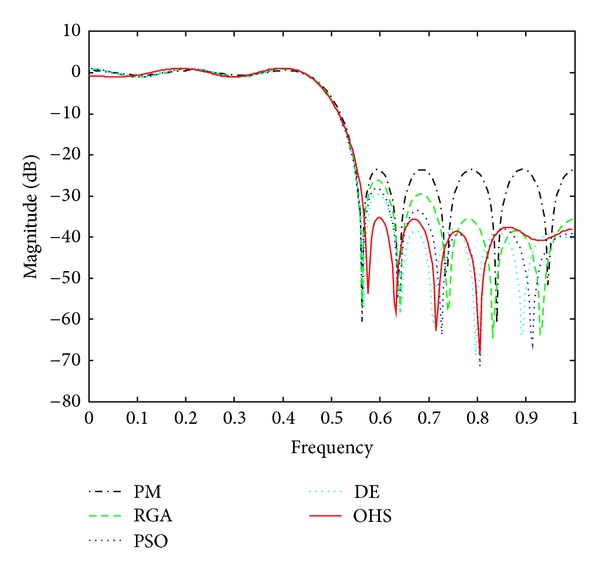
dB plots for the FIR LP filter of order 20.

**Figure 2:**
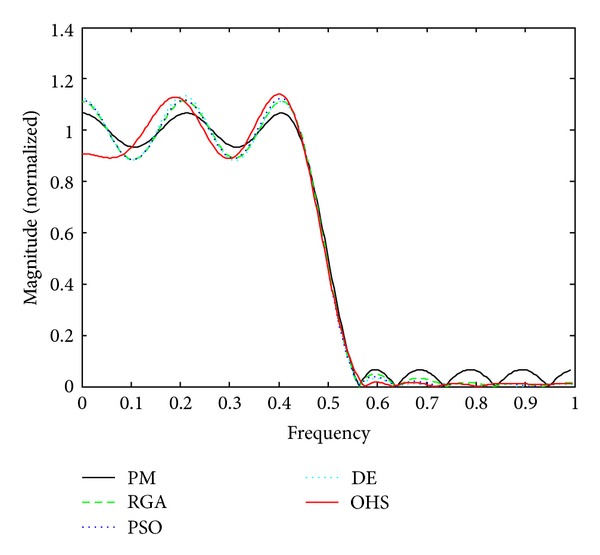
Normalized plots for the FIR LP filter of order 20.

**Figure 3:**
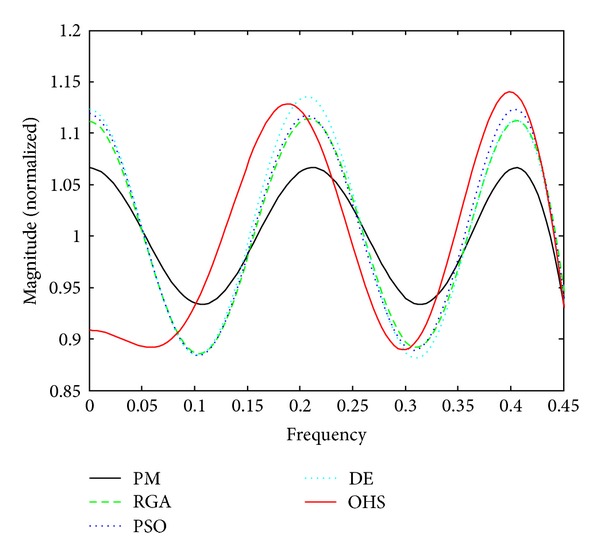
Normalized pass band ripple plots for the FIR LP filter of order 20.

**Figure 4:**
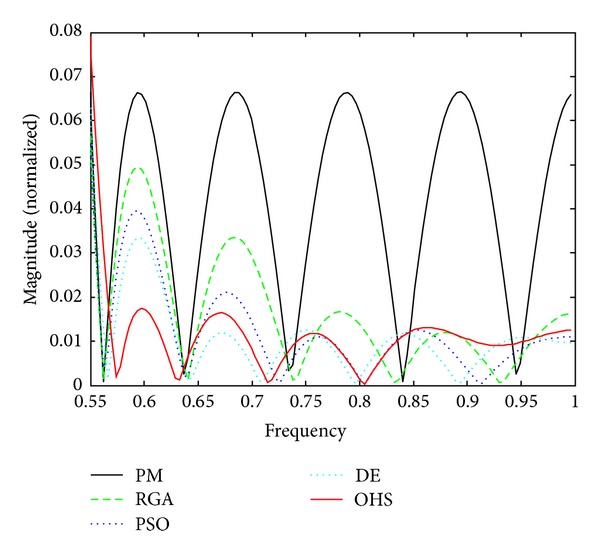
Normalized stop band ripple plots for the FIR LP filter of order 20.

**Figure 5:**
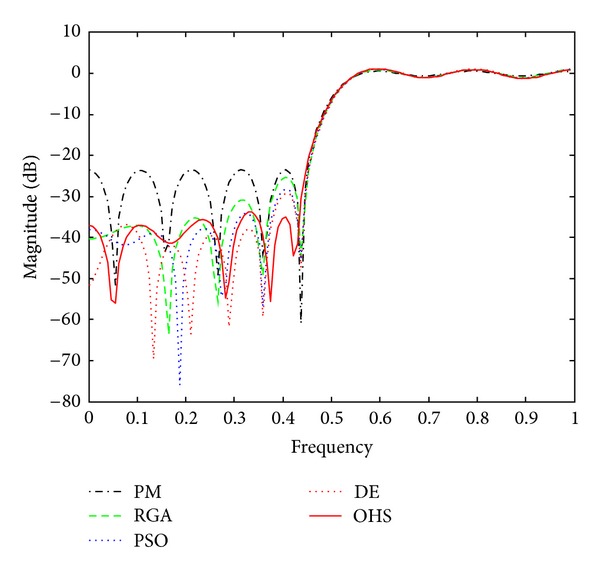
dB plots for the FIR HP filter of order 20.

**Figure 6:**
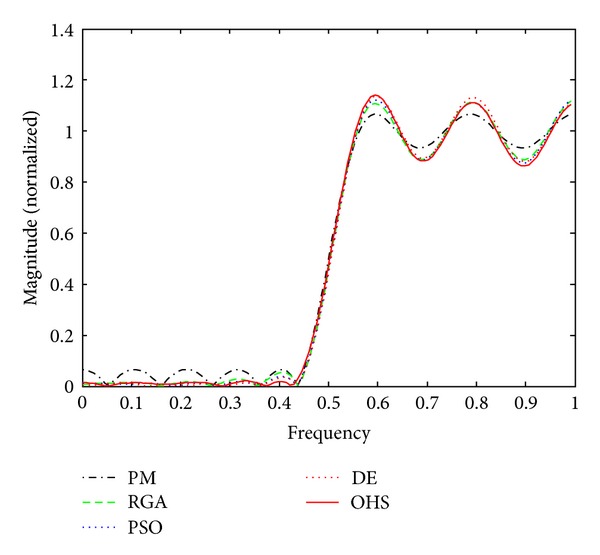
Normalized plots for the FIR HP filter of order 20.

**Figure 7:**
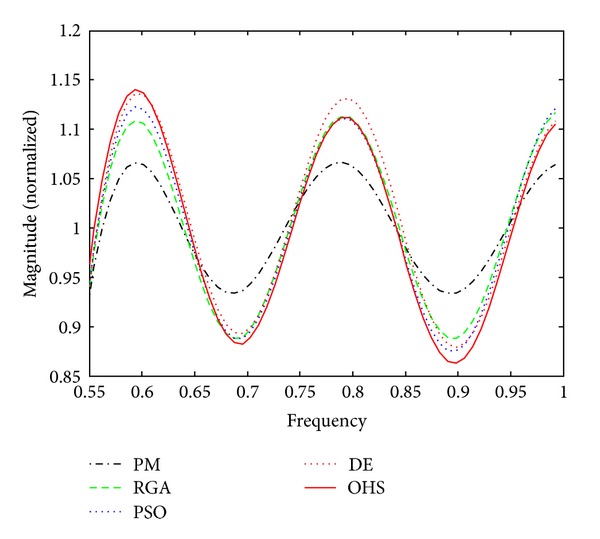
Normalized pass band ripple plots for the FIR HP filter of order 20.

**Figure 8:**
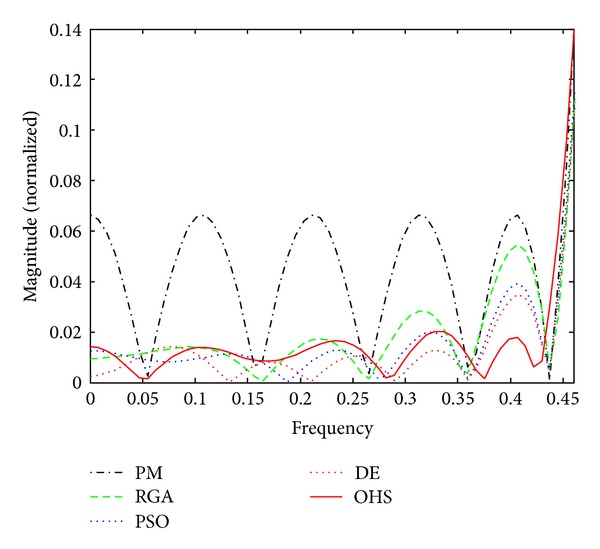
Normalized stop band ripple plots for the FIR HP filter of order 20.

**Figure 9:**
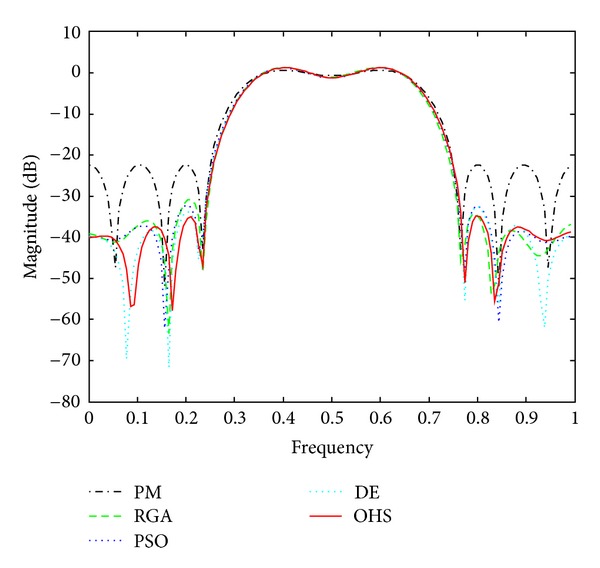
dB plots for the FIR BP filter of order 20.

**Figure 10:**
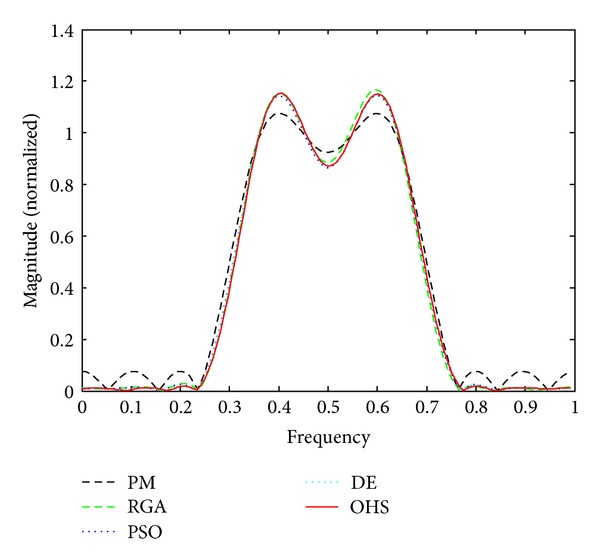
Normalized plots for the FIR BP filter of order 20.

**Figure 11:**
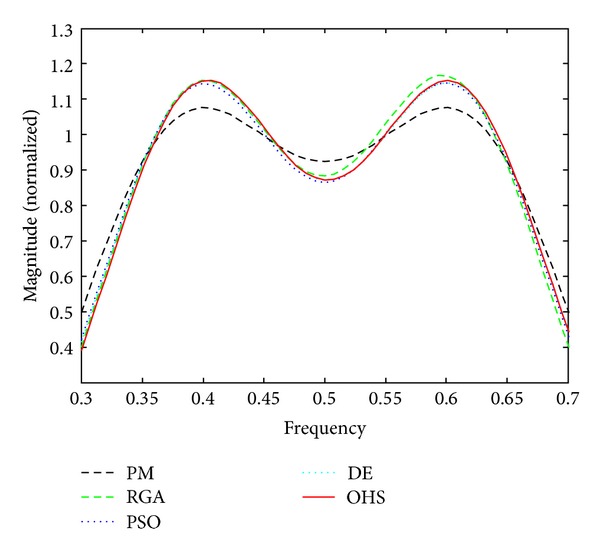
Normalized pass band ripple plots for the FIR BP filter of order 20.

**Figure 12:**
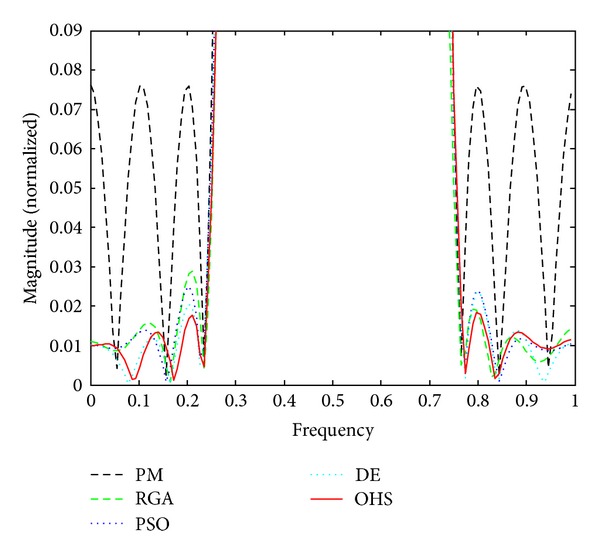
Normalized stop band ripple plots for the FIR BP filter of order 20.

**Figure 13:**
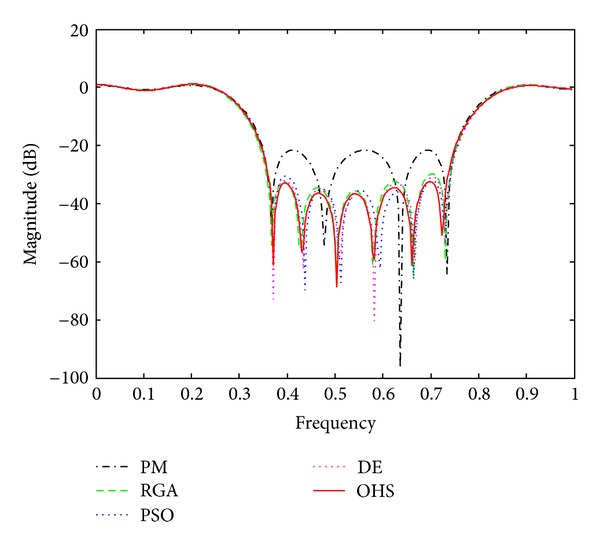
dB plots for the FIR BS filter of order 20.

**Figure 14:**
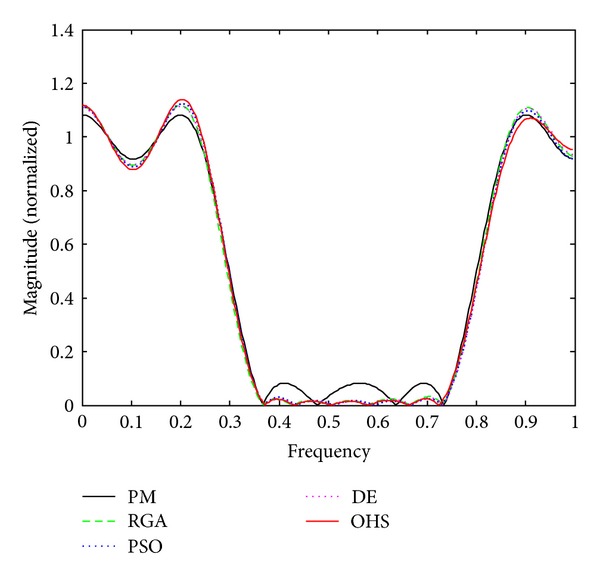
Normalized plots for the FIR BS filter of order 20.

**Figure 15:**
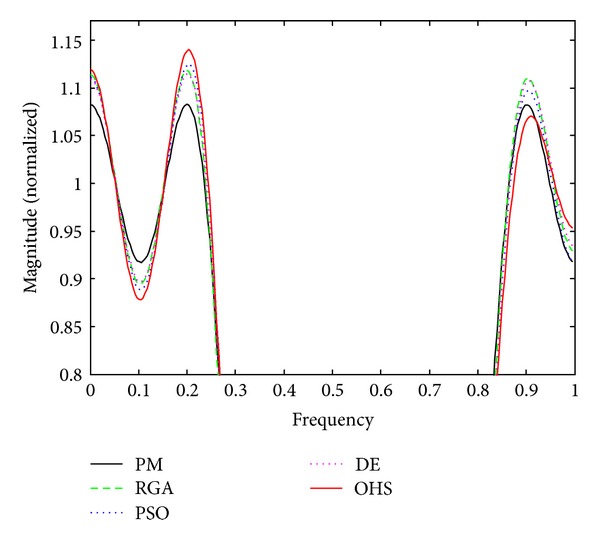
Normalized pass band ripple plot for the FIR BS filter of order 20.

**Figure 16:**
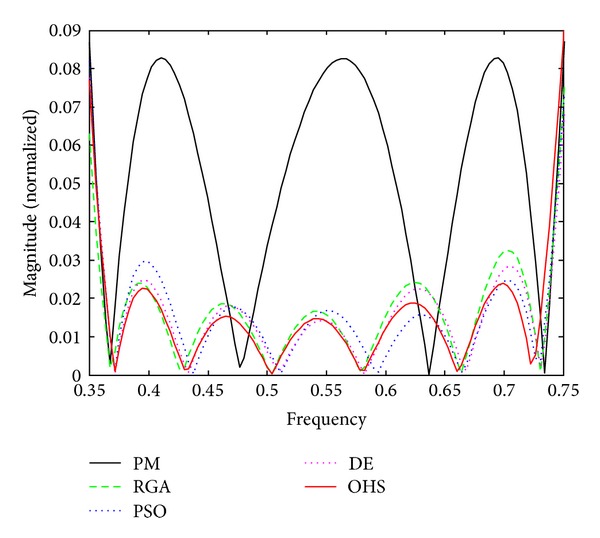
Normalized stop band ripple plot for the FIR BS filter of order 20.

Figures 1–4 show the magnitude responses of the 20th-order LP filter in various forms using PM, RGA, PSO DE, and OHS, respectively. The magnitude responses in dB are plotted in Figure 1 for the same. The normalized magnitude responses are shown in Figure 2.  Figure 3 shows normalized pass band ripple plots.  Figure 4 shows normalized stop band ripple plots. Figures 5, 6, 7, 8, 9, 10, 11, 12, 13, 14, 15, and 16 show magnitude responses in dB, normalized magnitude responses, normalized pass band ripples, and normalized stop band ripples for HP, BP, and BS filters, respectively. From the above figures and tables, it is observed that OHS results in better magnitude responses (dB), better normalized magnitude responses, and better normalized stop band ripple plots for all filters, as compared to those of PM, RGA, PSO, and DE.

### 4.1. Comparative Effectiveness and Convergence Profiles of RGA, PSO, DE and OHS Algorithms

**Figure 17:**
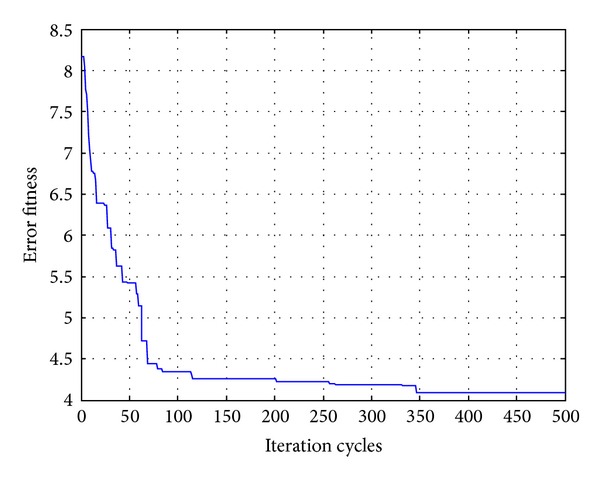
Convergence profile for RGA in case of FIR HP filter of order 20.

**Figure 18:**
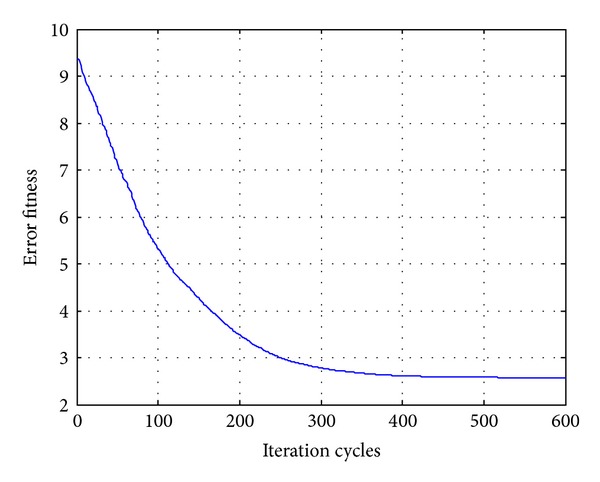
Convergence profile for PSO in case of FIR HP filter of order 20.

**Figure 19:**
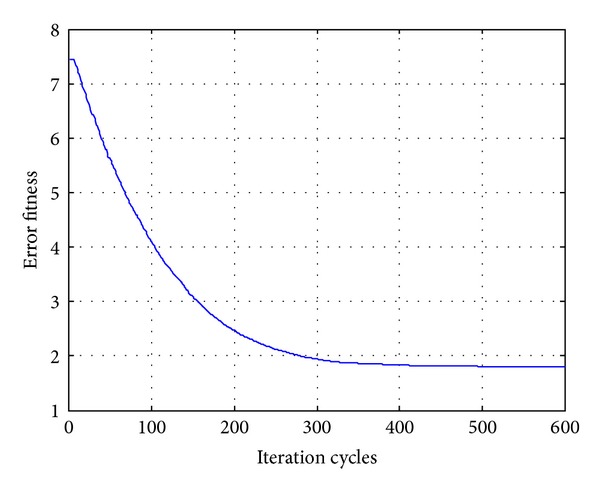
Convergence profile for DE in case of FIR HP filter of order 20.

**Figure 20:**
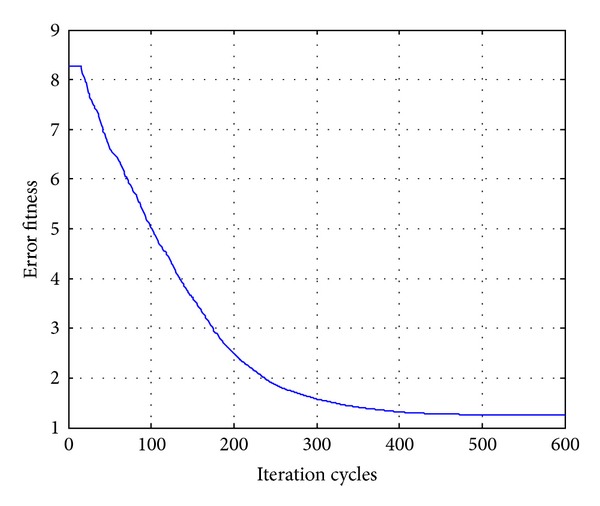
Convergence profile for OHS in case of FIR HP filter of order 20.

In order to compare the algorithms in terms of the error fitness values, the convergences of error fitness values obtained for the HP filter of order 20 for RGA, PSO, DE, and OHS are plotted as shown in Figures 17, 18, 19, and 20. Similar plots have also been obtained for the LP, BP, and BS filters of the same order, which are not shown here. OHS converges to much lower error fitness value as compared to RGA, PSO, and DE which yield suboptimal higher values of error fitness values. RGA converges to the minimum error fitness value of 4.088 in 18.676116 s; PSO converges to the minimum error fitness value of 2.575 in 17.2266628 s; DE converges to the minimum error fitness value of 1.803 in 17.3641545 s, whereas OHS converges to the minimum error fitness value of 1.248 in 18.96705 s.

For all types of filters, OHS converges to the least minimum error fitness values in finding the optimum filter coefficients in moderately less execution times, which may be verified from Tables 7, 8, 9, and 10. With a view to the above fact, it may be inferred that the performance of OHS is the best among all algorithms. All optimization programs were run in MATLAB 7.5 version on core (TM) 2 duo processor, 3.00 GHz with 2 GB RAM.

## 5. Conclusion

In this paper, a novel opposition-based harmony search (OHS) algorithm is applied to the solution of the constrained, multimodal FIR filter design problem, yielding optimal filter coefficients. Comparison of the results of PM, RGA, PSO, DE, and OHS algorithm has been made. It is revealed that OHS has the ability to converge to the best quality near optimal solution and possesses the best convergence characteristics in moderately less execution times among the algorithms. The simulation results clearly indicate that OHS demonstrates better performance in terms of magnitude response, minimum stop band ripple, and maximum stop band attenuation with a very little deterioration in the transition width. Thus, OHS may be used as a good optimizer for obtaining the optimal filter coefficients in any practical digital filter design problem of digital signal processing systems.

## Figures and Tables

**Algorithm 1:**
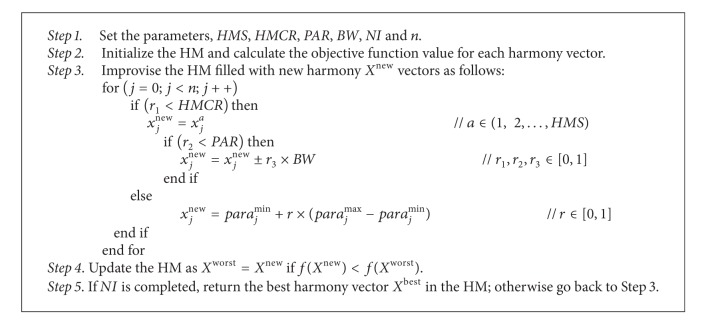
HS Algorithm.

**Algorithm 2:**
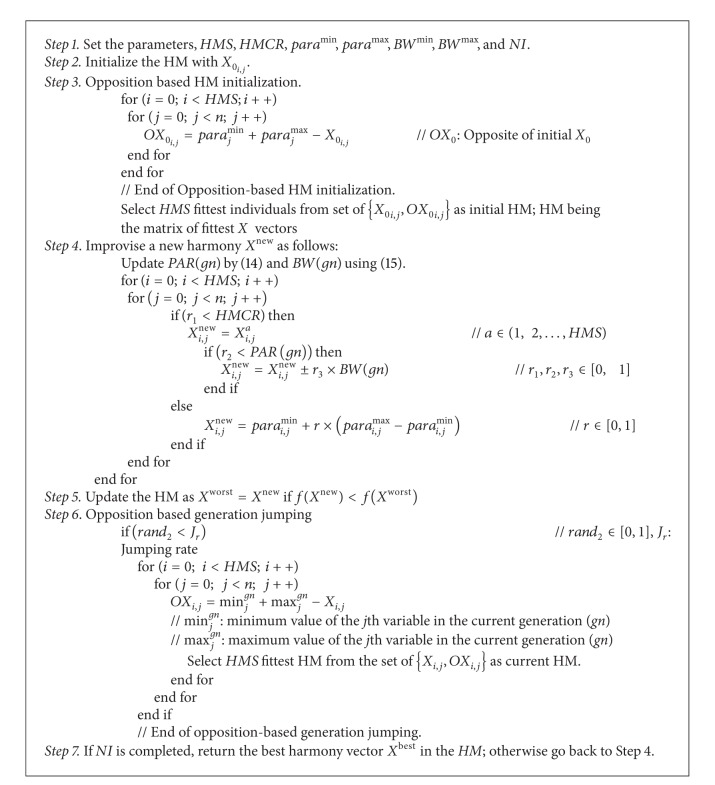
OHS Algorithm.

**Table 1:** Control parameters of RGA, PSO, DE and OHS.

RGA	PSO	DE	OHS
Population size = 120, iteration cycles = 600, crossover rate = 1, crossover = two point crossover, mutation rate = 0.01, mutation = Gaussian mutation, and selection = roulette	Population size = 120, iteration cycles = 600, *C*_1_ = *C*_2_ = 2.05, *v*_*i*_^min⁡^ = 0.01, *v*_*i*_^max⁡^ = 1.0, *w*_max⁡_ = 1.0, and *w*_min⁡_ = 0.4	Population size = 120; iteration cycles = 600; *C*_*r*_ = 0.3; *F* = 0.5	Population size = 120; iteration cycles = 600; *HMCR* = 0.6, *PAR*_min_ = 0; *PAR*_max_ = 0.9; *BW*_min_ = 0.000001; and *BW*_max_ = 1

**Table 2:** Optimized coefficients of the FIR LP filter of order 20.

*h*(*N*)	RGA	PSO	DE	OHS
*h*(1) = *h*(21)	0.020644508012550	0.025116793352393	0.027005399982491	0.020509263646238
*h*(2) = *h*(20)	0.048721413185106	0.047219259300299	0.047266866797926	0.032132175858325
*h*(3) = *h*(19)	0.005868601564964	0.003546242723169	0.005320204222841	−0.013117080938103
*h*(4) = *h*(18)	−0.040966865300227	−0.040094047283599	−0.038982294859373	−0.053943547883771
*h*(5) = *h*(17)	−0.000863506780022	−0.000520432067214	−0.003452235386096	−0.012003255218944
*h*(6) = *h*(16)	0.059796031265565	0.060907207778672	0.057946858872171	0.048106434177123
*h*(7) = *h*(15)	−0.001408842862974	−0.001759240756773	−0.002051400593964	−0.007969148591832
*h*(8) = *h*(14)	−0.103117834700311	−0.103613994946693	−0.102715267629915	−0.113260035845183
*h*(9) = *h*(13)	−0.000440644382089	0.000627623037422	0.001692937801793	−0.007125766240178
*h*(10) = *h*(12)	0.317600651261946	0.318119036548684	0.319795676258768	0.310972208736947
*h*(11)	0.500018538901556	0.500018538901556	0.500018538901556	0.500018538901555

**Table 3:** Optimized coefficients of the FIR HP filter of order 20.

*h*(*N*)	RGA	PSO	DE	OHS
*h*(1) = *h*(21)	0.021731353326545	0.025559145974814	0.029041921147266	0.027958875232026
*h*(2) = *h*(20)	−0.048131602227058	−0.047413653181042	−0.045873202416582	−0.046219133874889
*h*(3) = *h*(19)	0.006298189918824	0.005135430273491	0.002950561225606	0.005908752612340
*h*(4) = *h*(18)	0.041895345956760	0.039988099089174	0.041311799862169	0.041684671886351
*h*(5) = *h*(17)	0.000879943669486	0.001405996354021	−0.000283997158910	0.000484971329494
*h*(6) = *h*(16)	−0.059027866591514	−0.060283192968605	−0.060002355552046	−0.058130379060402
*h*(7) = *h*(15)	−0.000013559660394	0.000768613197325	−0.003921102337490	0.003431061619144
*h*(8) = *h*(14)	0.104257677520726	0.105120739785348	0.106119151142982	0.105123375775675
*h*(9) = *h*(13)	0.003823743541217	0.001471927911810	−0.000565063060302	−0.007107362649439
*h*(10) = *h*(12)	−0.316631427282300	−0.315471590838371	−0.320083906578923	−0.316061867104696
*h*(11)	0.499468012025621	0.499981461098444	0.499981461098444	0.499981461098444

**Table 4:** Optimized coefficients of the FIR BP filter of order 20.

*h*(*N*)	RGA	PSO	DE	OHS
*h*(1) = *h*(21)	0.028502857888104	0.024910907374264	0.024315759656957	0.026978129356347
*h*(2) = *h*(20)	−0.001893868108392	0.000092972958187	0.002788616388318	0.003014521009408
*h*(3) = *h*(19)	−0.076189026154460	−0.074535581888545	−0.075883731240533	−0.075573145593866
*h*(4) = *h*(18)	0.000994123920259	−0.000579129089510	−0.003313368788351	−0.005667273096237
*h*(5) = *h*(17)	0.053196793860741	0.058322287561503	0.056134992376798	0.054764314739432
*h*(6) = *h*(16)	−0.000639149080848	−0.000187613541059	0.000257826174027	−0.001285546925313
*h*(7) = *h*(15)	0.100057194730152	0.093164875388599	0.090912344142406	0.093422699573072
*h*(8) = *h*(14)	0.001409980793664	0.001012723950710	0.002199187065772	0.003282759787987
*h*(9) = *h*(13)	−0.299380312728113	−0.296866917983546	−0.300934749358008	−0.299362912756530
*h*(10) = *h*(12)	−0.000752480372393	−0.000392232750468	−0.001799401229551	−0.004731509416213
*h*(11)	0.400369877077545	0.400369877077545	0.400369877077545	0.400369877077545

**Table 5:** Optimized coefficients of the FIR BS filter of order 20.

*h*(*N*)	RGA	PSO	DE	OHS
*h*(1) = *h*(21)	0.008765244188382	0.005065078955931	0.005738163937772	0.011156843719480
*h*(2) = *h*(20)	0.054796923249762	0.054496716662981	0.053905628215447	0.052282877968109
*h*(3) = *h*(19)	0.001796419983890	0.005809988516188	0.002902448937586	0.009942391820387
*h*(4) = *h*(18)	0.048911654246731	0.051144048751957	0.049349878942931	0.047219028225649
*h*(5) = *h*(17)	−0.054718457691943	−0.050663949788261	−0.050884656047053	−0.049531151275131
*h*(6) = *h*(16)	−0.060963142228236	−0.062741465298722	−0.063088550820316	−0.064382546432969
*h*(7) = *h*(15)	0.004293459264617	−0.000062718416445	0.004089341810059	−0.000512890612780
*h*(8) = *h*(14)	−0.065342448643273	−0.068916923681426	−0.068023108311494	−0.067902719326839
*h*(9) = *h*(13)	0.300682045893488	0.297478557865240	0.299063386928411	0.296806655163297
*h*(10) = *h*(12)	0.069036675664641	0.074390206250426	0.071701365941159	0.074321537635153
*h*(11)	0.499582536276171	0.499582536276171	0.500000357523254	0.500000357523254

**Table 6:** Comparison of stop band attenuations for different types of FIR filters, each of order 20 using different algorithms.

Filter type	Maximum stop band attenuation (dB)				
	PM	RGA	PSO	DE	OHS
LP	23.54	26.11	28.03	29.53	35.16
HP	23.55	25.25	28.10	29.16	33.86
BP	22.38	30.80	32.03	32.58	34.76
BS	21.65	29.73	30.56	30.96	32.45

**Table 7:** Other comparative results of performance parameters of all algorithms for the FIR LP filter of order 20.

Algorithm	FIR LP filter of order 20		
	Maximum, average stop band ripple (normalized)	Transition width (normalized)	Execution time for 100 cycles (s)
PM	0.06651, 0.066282	0.0838	—
RGA	0.04949, 0.025620	0.0853	5.7174
PSO	0.03967, 0.019052	0.0869	3.3286
DE	0.03339, 0.076192	0.0908	3.9543
OHS	0.01746, 0.045708	0.0994	3.8321

**Table 8:** Other comparative results of performance parameters of all algorithms for the FIR HP filter of order 20.

Algorithm	FIR HP filter of order 20		
	Maximum, average stop band ripple (normalized)	Transition width (normalized)	Execution time for 100 cycles (s)
PM	0.06645, 0.06637	0.0839	—
RGA	0.05461, 0.02860	0.0864	5.3667
PSO	0.03935, 0.01916	0.0867	3.04358
DE	0.03483, 0.01611	0.0878	3.93745
OHS	0.02027, 0.01651	0.1004	3.79341

**Table 9:** Other comparative results of performance parameters of all algorithms for the FIR BP filter of order 20.

Algorithm	FIR BP filter of order 20		
	Maximum, average stop band ripple (normalized)	Transition width (normalized)	Execution time for 100 cycles
PM	0.07609, 0.076017	0.0875	—
RGA	0.02885, 0.016855	0.0945	6.3827
PSO	0.02504, 0.015893	0.1009	4.6850
DE	0.02350, 0.015125	0.0987	4.9832
OHS	0.01828, 0.01408	0.0988	4.7156

**Table 10:** Other comparative results of performance parameters of the FIR BS filter of order 20 for all algorithms.

Algorithm	FIR BS filter of order 20		
	Maximum, average stop band ripple (normalized)	Transition width (normalized)	Execution time for 100 cycles
PM	0.08273, 0.08268	0.0905	—
RGA	0.03262, 0.02322	0.0959	6.2846
PSO	0.02966, 0.02092	0.0936	4.8777
DE	0.02832, 0.02161	0.0981	5.0005
OHS	0.02385, 0.01906	0.1069	4.9943

**Table 11:** Statistical parameters of FIR LP filters for different algorithms.

Algorithm	FIR LP filter of order 20							
	Pass band ripple (normalized)				Stop band attenuation (dB)			
	Maximum	Mean	Variance	Standard deviation	Maximum	Mean	Variance	Standard deviation
PM	0.0664	0.06616	3.84*e* − 8	0.000196	23.54	23.572	0.000696	0.026382
RGA	0.1142	0.11224	5.11*e* − 6	0.002261	26.11	33.056	20.5999	4.538712
PSO	0.1230	0.11714	1.55*e* − 5	0.003939	28.03	35.588	18.6021	4.313015
DE	0.1360	0.12152	6.56*e* − 5	0.008099	29.53	36.784	13.30738	3.647929
OHS	0.1400	0.12195	17.3*e* − 5	0.013171	35.16	37.014	1.804384	1.343274

**Table 12:** Statistical parameters of FIR HP filters for different algorithms.

Algorithm	FIR HP filter of order 20							
	Pass band ripple (normalized)				Stop band attenuation (dB)			
	Maximum	Mean	Variance	Standard deviation	Maximum	Mean	Variance	Standard deviation
PM	0.0663	0.06612	2.16*e* − 8	0.000147	23.55	23.560	0.00012	0.010954
RGA	0.1170	0.11262	6.64*e* − 6	0.002577	25.25	32.110	20.59505	4.538177
PSO	0.1249	0.11820	3.12*e* − 5	0.005590	28.1	35.396	16.09082	4.011337
DE	0.1360	0.12060	0.000137	0.011693	29.16	37.058	18.3217	4.280385
OHS	0.1400	0.12228	0.000191	0.013819	33.86	35.728	1.474056	1.214107

**Table 13:** Statistical parameters of FIR BP filters for different algorithms.

Algorithm	FIR BP filter of order 20							
	Pass band ripple (normalized)				Stop band attenuation (dB)			
	Maximum	Mean	Variance	Standard deviation	Maximum	Mean	Variance	Standard deviation
PM	0.0763	0.07610	2*e* − 8	0.000141	22.38	22.42	0.007267	0.085245
RGA	0.1670	0.145167	0.000448	0.021175	30.80	35.941667	7.752181	2.784278
PSO	0.1460	0.141367	2.11*e* − 5	0.004597	32.03	36.6	10.40607	3.225844
DE	0.1520	0.142533	0.000101	0.010062	32.58	36.86	7.605033	2.757722
OHS	0.1530	0.144567	0.000126	0.011227	34.76	37.215	3.209158	1.791412

**Table 14:** Statistical parameters of FIR BS filters for different algorithms.

Algorithm	FIR BS filter of order 20							
	Pass band ripple (normalized)				Stop band attenuation (dB)			
	Maximum	Mean	Variance	Standard deviation	Maximum	Mean	Variance	Standard deviation
PM	0.083	0.08272	1.54*e* − 7	0.000392	21.65	21.653333	2.22*e* − 5	0.004714
RGA	0.118	0.1037	0.000285	0.016869	29.73	32.924	4.148024	2.03667
PSO	0.125	0.10554	0.000221	0.014868	30.56	33.858	4.566376	2.136908
DE	0.115	0.10024	0.000322	0.017937	30.96	33.552	4.405096	2.098832
OHS	0.140	0.09952	0.00123	0.035072	32.45	34.564	2.914104	1.707075

**Table 15:** Comparison of OHS-based results with other reported results.

Model	Parameter					
	Filter type	Order	Maximum stop band attenuation (dB)	Maximum pass band ripple (normalized)	Maximum stop band ripple (normalized)	Transition width
Oliveira et al. [15]	Band pass	30	<33 dB	NR*	NR*	>0.1
Karaboga and Cetinkaya [18]	Low pass	20	NR*	>0.08	>0.09	>0.16
Liu et al. [19]	Low Pass	20	NR*	0.04	>0.07	>0.06
Najjarzadeh and Ayatollahi [21]	Low pass	33	<29 dB	NR*	NR*	NR*
	Band pass	33	<25 dB	NR*	NR*	NR*
Ababneh and Bataineh [23]	Low pass	30	<30 dB (Approx.)	0.15	0.031	0.05
Sarangi et al. [26]	Low pass	20	<27 dB	>0.1	>0.06	>0.15
	Band pass	20	<8 dB	>0.2	>0.05	>0.07
Mondal et al. [30]	High pass	20	34.03	0.129	0.02392	0.0825
Luitel and Venayagamoorthy [33]	Low pass	20	<27 dB	0.291	0.270	>0.13
OHS	Low pass	20	35.16	0.140	0.01746	0.0994
	High pass	20	33.86	0.140	0.02027	0.1004
	Band pass	20	34.76	0.153	0.01828	0.0988
	Band stop	20	32.45	0.140	0.02385	0.1069

*NR means not reported in the referred literature.
